# Emergency Lifesaving Management of a Homicidal Cut-Throat Injury: A Case Report

**DOI:** 10.7759/cureus.47789

**Published:** 2023-10-27

**Authors:** Koshy Varghese, Prashant Sharma, Seelora Sahu, Abhijit Kumar, Gunjan Wadhwa

**Affiliations:** 1 Department of Anaesthesiology, Tata Main Hospital, Jamshedpur, IND; 2 Department of Anaesthesiology, Manipal Tata Medical College, Jamshedpur, IND; 3 Department of Otolaryngology, Raj Hospital, Ranchi, IND

**Keywords:** severed trachea, difficult airway intubation, emergency airway management, homicidal, cut-throat injury

## Abstract

Patients with cut-throat injuries presenting to the emergency department pose a serious challenge and often require a multidisciplinary mode of management. The role of an anesthesiologist is primarily airway management, either by endotracheal intubation or tracheostomy. Securing a definitive airway before wound exploration and repair of transected tissues is difficult as such injuries are often accompanied by distortion of the airway anatomy complicated by vascular or tissue bleed. Here, we report a case of a homicidal cut-throat injury in a 55-year-old female who was bleeding profusely from the neck, along with airway compromise in the form of a transected trachea. Timely intervention by a multidisciplinary team consisting of anesthesiologists and otorhinolaryngologists resulted in a favorable outcome.

## Introduction

Cut-throat wounds are a well-recognized method of homicide, a less commonly used mode of suicide, and are rarely accidental [1]. If not treated timely, they may lead to the death of the patient due to asphyxia and hemorrhage [2]. Prevention of these complications depends on immediate resuscitation by securing the airway by tracheostomy or intubation, prompt control of hemorrhage, and blood replacement [3]. In this case, immediate airway management with intubation through the transected trachea, along with partial hemostasis, was achieved in the emergency department, which proved to be lifesaving until definitive surgical repair and tracheostomy were performed in the operating room (OR), where blood and blood products could be procured.

## Case presentation

The emergency department paged the anesthesiologist for the urgent airway management of a 55-year-old female patient with a homicidal cut-throat injury. On examination, the patient was bleeding profusely through the front of the neck wound and was extremely restless and poorly responsive to commands. She was in hypovolemic shock with a blood pressure of 80/64 mmHg, heart rate of 150 beats per minute, and respiratory rate of 26 breaths per minute with SpO_2_ of 65-70% even with 10 L/minute of oxygen through a mask.

**Figure 1:**
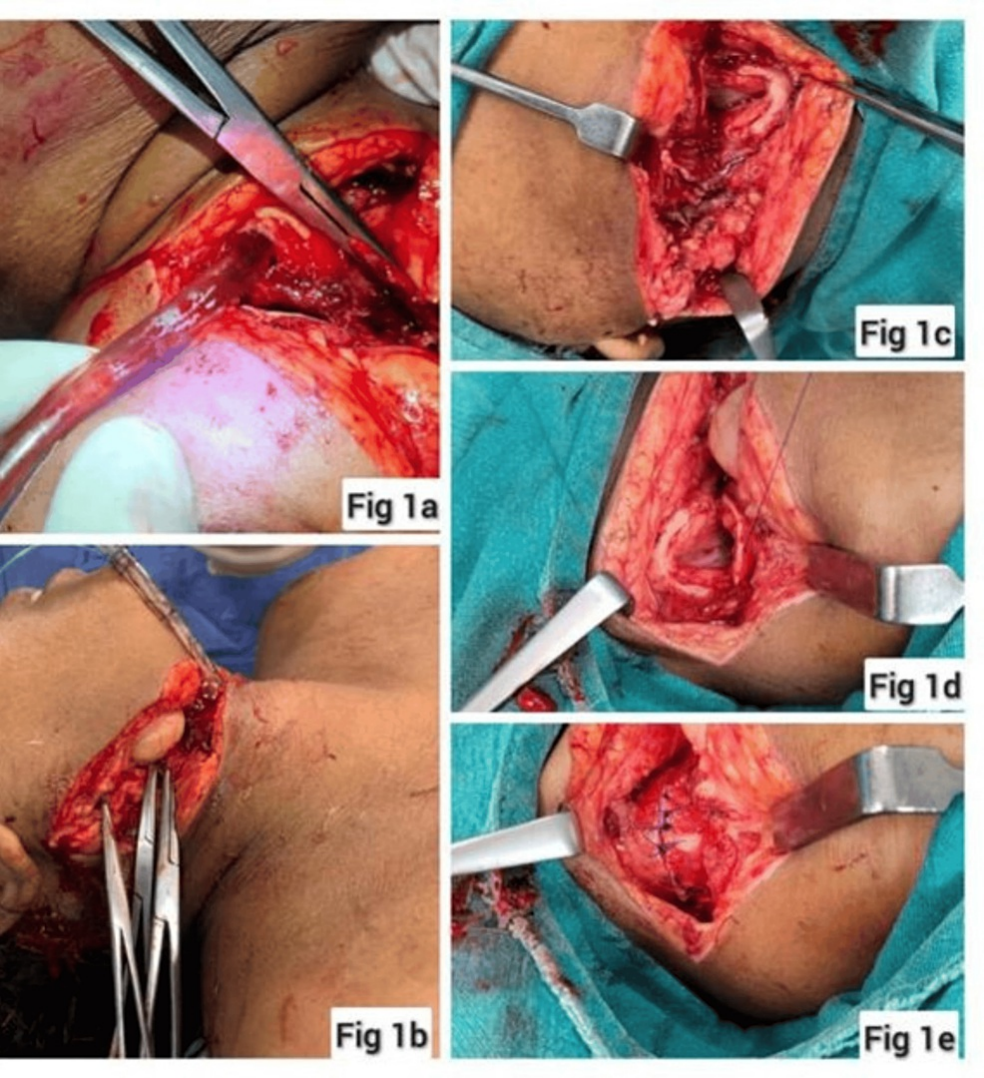
(a) Endotracheal tube inserted through the distal cut end of the trachea. (b) Endotracheal tube secured with sutures and bleeding controlled by application of artery forceps. (c) The distal end of the open tracheal lacerated wound. (d and e) Repair of the severed trachea and bleeding vessels.

The patient was oxygenated with 100% oxygen through Bain’s circuit, was sedated with midazolam, and laryngoscopic intubation was attempted to secure the airway; however, this had to be aborted in view of profuse bleeding into the oropharynx and further desaturation. Hence, oxygenation was recommenced after suctioning the blood from the airway. The surgical team tried to control the neck wound bleed with suctioning, surgical mops, and the application of artery forceps over the superficially visible bleeding points. However, blood kept welling up from the deeper planes because of which the injured critical neck structures including the tracheal source of air leak (visible air bubbling through the bloody pool) could not be identified. Aided by continuous suction of the welling blood pool, a general direction of the source of the air leak was located. Subsequently, on finger palpation around this area, an opening that felt like a tracheal lumen could be appreciated just below the thyroid cartilage, through which a gum elastic bougie was blindly and gently guided till it touched what felt like the carina. An endotracheal tube of size 7.0 mm internal diameter was railroaded over it (Figure 1). The bougie was then withdrawn and an endotracheal tube was connected to Bain’s circuit with oxygen at 10 L/minute. Spontaneous movement of the breathing bag with the patient’s respiration was noted, there was bilateral air entry, and oxygen saturation improved to 100%. Restlessness decreased and pressure could now be applied around the trachea to localize and better control deeper plane bleeders with artery forceps. The endotracheal tube was fixed to the chin with sutures (Figure 1). Fluid resuscitation was initiated with colloids and Ringer’s lactate with two 16G cannulas secured simultaneously.

The patient was shifted to the OR. Her hemodynamic parameters at this stage improved, with a blood pressure of 100/70 mmHg, heart rate of 115 beats/minute, and SpO_2_ of 100%. Induction of anesthesia was done with ketamine 75 mg, midazolam 1 mg, fentanyl 100 µg, and vecuronium 6 mg and maintained with oxygen, nitrous oxide, and sevoflurane on volume control mode. The right femoral vein was cannulated, and adrenaline infusion at 0.1 µg/kg/minute was started. She was transfused with four fresh frozen plasma, four packed red blood cells, and four platelet concentrates, and, subsequently, the hemodynamic parameters improved.

The patient was then positioned with a neck extension for a tracheostomy. Once the tracheal stoma was made, the endotracheal tube was withdrawn above this level and the trachea was cannulated with a 7.5 Fr cuffed tracheostomy tube and the placement was confirmed with end-tidal carbon dioxide waveform.

Thereafter, the injured trachea and major vessels were repaired, a nasogastric feeding tube was placed, and the primary wound was closed. Neuromuscular blockade was reversed, and the patient was shifted to a high-dependency unit and ventilated in pressure-regulated volume control mode.

**Figure 2:**
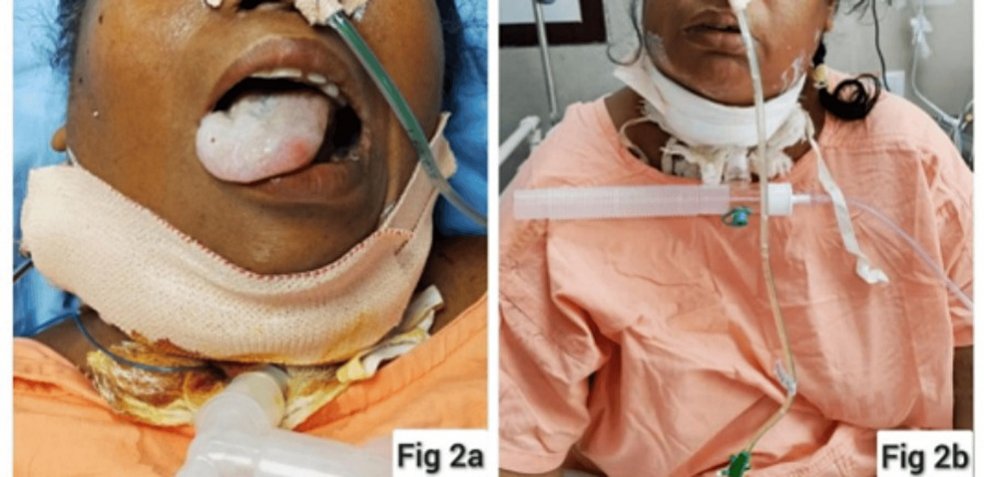
The patient after being shifted to the ward with a tracheostomy tube in situ.

She was tapered off inotropes once her hemodynamical parameters were stable and blood gas reports were acceptable. She was weaned off the ventilator and put on a T-piece with oxygen support. It was noticed that she had a transient deviation of her tongue to the left which later improved (Figure 2). The patient was subsequently decannulated after two weeks. She could cough out and swallow nicely, could phonate with good voice quality, and was successfully discharged on the 17th postoperative day.

## Discussion

Homicidal cut-throat injuries are inflicted by sharp items, and the wound may be incised or simulated as incised injuries. Although not uncommon, there is a paucity of literature about its incidence and survival rate [4]. The speed of shifting safely from the street to the operation theater is the cornerstone in the management of such injuries [5].

Such injuries are associated with airway compromise and injury to the major vessels and nerves of the neck, as well as other structures such as cartilage and glands. Hence, these injuries are potentially life-threatening. Rendering airway management is a crucial factor in such patients and must be given priority [5].

In this case, the patient reached the emergency room in shock with profuse bleeding and desaturation due to a probably injured airway. Securing the airway was the priority. An initial laryngoscopy was attempted, which resulted in even more profuse bleeding into the oropharynx and had to be aborted. Instead, her spontaneous respiration was supplemented with 100% oxygen through Bain’s circuit after suctioning the blood from the oropharynx. An attempt was made to control bleeding with the application of artery forceps over the superficially visible bleeding points allowing us to notice the bubbling of air through the blood pool which guided us to the hidden partially cut end of the trachea. A simultaneous effort to control the bleeding by the application of artery forceps or vascular clamps should always be made to stop exsanguination and more importantly for early localization of any open tracheal injury [6]. There are reports where the endotracheal tube was inserted through the tracheal opening, and this can be used as an emergency alternative route to secure the airway until a definitive airway can be planned.

Once the airway was secured in this manner, the anatomy became clearer, and better control of bleeding with pressure using surgical mops became possible. This helped in identifying and applying artery forceps to the deeper bleeding vessels. Now, with the airway secured, better bleeding control, and ongoing fluid resuscitation, the patient could be safely shifted to the OR for tracheostomy, wound exploration, and definitive repair. Quick thinking, rapid resuscitation, and a definitive surgical repair led to a faster improvement of hemodynamic parameters and early weaning from ventilatory support.

## Conclusions

Challenges in the management of cut-throat injuries in the emergency department range from airway management to the control of ongoing blood loss and subsequent hemorrhagic shock. Timely intervention and coordination between specialties in this case of homicidal cut-throat injury resulted in an excellent outcome with the patient discharged in a stable condition.
